# Evaluating Community-Facing Virtual Modalities to Support Complex Neurological Populations During the COVID-19 Pandemic: Protocol for a Mixed Methods Study

**DOI:** 10.2196/28267

**Published:** 2021-07-23

**Authors:** Katelyn Brehon, Jay Carriere, Katie Churchill, Adalberto Loyola-Sanchez, Petra O'Connell, Elisavet Papathanassoglou, Rob MacIsaac, Mahdi Tavakoli, Chester Ho, Kiran Pohar Manhas

**Affiliations:** 1 School of Public Health University of Alberta Edmonton, AB Canada; 2 Department of Electrical and Computer Engineering University of Alberta Edmonton, AB Canada; 3 Department of Occupational Therapy Faculty of Rehabilitation Medicine University of Alberta Edmonton, AB Canada; 4 Department of Medicine Division of Physical Medicine and Rehabilitation University of Alberta Edmonton, AB Canada; 5 Obesity Diabetes Nutrition Strategic Clinical Network Alberta Health Services Calgary, AB Canada; 6 Neurosciences, Rehabilitation & Vision Strategic Clinical Network Alberta Health Services Calgary, AB Canada; 7 Spinal Cord Injury Alberta Edmonton, AB Canada

**Keywords:** telehealth, evaluation, rehabilitation, musculoskeletal, neurological, COVID-19, spinal cord injury, advice line, webinar, artificial intelligence, machine learning, community engagement

## Abstract

**Background:**

The COVID-19 pandemic and concomitant governmental responses have created the need for innovative and collaborative approaches to deliver services, especially for populations that have been inequitably affected. In Alberta, Canada, two novel approaches were created in Spring 2020 to remotely support patients with complex neurological conditions and rehabilitation needs. The first approach is a telehealth service that provides wayfinding and self-management advice to Albertans with physical concerns related to existing neurological or musculoskeletal conditions or post-COVID-19 recovery needs. The second approach is a webinar series aimed at supporting self-management and social connectedness of individuals living with spinal cord injury.

**Objective:**

The study aims to evaluate the short- and long-term impacts and sustainability of two virtual modalities (telehealth initiative called Rehabilitation Advice Line [RAL] and webinar series called Alberta Spinal Cord Injury Community Interactive Learning Seminars [AB-SCILS]) aimed at advancing self-management, connectedness, and rehabilitation needs during the COVID-19 pandemic and beyond.

**Methods:**

We will use a mixed-methods evaluation approach. Evaluation of the approaches will include one-on-one semistructured interviews and surveys. The evaluation of the telehealth initiative will include secondary data analyses and analysis of call data using artificial intelligence. The evaluation of the webinar series will include analysis of poll questions collected during the webinars and YouTube analytics data.

**Results:**

The proposed study describes unique pandemic virtual modalities and our approaches to evaluating them to ensure effectiveness and sustainability. Implementing and evaluating these virtual modalities synchronously allows for the building of knowledge on the complementarity of these methods. At the time of submission, we have completed qualitative and quantitative data collection for the telehealth evaluation. For the webinar series, so far, we have distributed the evaluation survey following three webinars and have conducted five attendee interviews.

**Conclusions:**

Understanding the impact and sustainability of the proposed telehealth modalities is important. The results of the evaluation will provide data that can be actioned and serve to improve other telehealth modalities in the future, since health systems need this information to make decisions on resource allocation, especially in an uncertain pandemic climate. Evaluating the RAL and AB-SCILS to ensure their effectiveness demonstrates that Alberta Health Services and the health system care about ensuring the best practice even after a shift to primarily virtual care.

**International Registered Report Identifier (IRRID):**

DERR1-10.2196/28267

## Introduction

### Background

The COVID-19 pandemic and concomitant governmental responses have created the need for innovative and collaborative approaches to deliver services, especially for populations that have been inequitably affected. The pandemic has triggered inconsistency in health service delivery and variable social distancing mandates, each fluctuating by time, geography, and social mores.

Vulnerable populations, such as individuals living with disabilities in the community, are at risk for negative health outcomes because they are unable to access necessary community resources during the COVID-19 pandemic [1,2]. It has been reported that 22% of Canadians have disabilities (eg, spinal cord injury and Parkinson disease) [3]. Mandated social distancing seen in the waves of COVID-19 has suspended clinics, homecare, and other support services, which are especially important for Canadians with disabilities. As services opened, virtual care and alternative service delivery formats offered opportunities and presented challenges for this population to socially connect and access the care needed. These challenges have put these patients at high risk for deterioration, possible hospitalization, and unnecessary emergency room visits. Extended isolation also exacerbates caregiver demands for people living with disabilities [4].

The influx of COVID-19 patients (with actual and expected surges) will introduce more patients requiring rehabilitation care into the system due to the physical, respiratory, and neurological symptoms after acute COVID-19 [5-11]. Early evidence suggests that patients with COVID-19 undergoing intensive care will likely experience functional decline due to organ dysfunction, deconditioning, cognitive changes, and psychological sequelae (depression and anxiety) for extended periods after hospitalization [5-7]. In addition to the patients experiencing post-COVID symptoms, there have been, and will continue to be, patients who present with physical and neurological sequelae unrelated to COVID-19, such as spinal cord injuries.

### Utilizing and Evaluating Telehealth Modalities

The pandemic has catalyzed the rapid adoption of telehealth practices to ensure the continuity of appropriate care during this time [12]. Telehealth initiatives adopted during the pandemic have included telephone helplines; virtual meetings using platforms like Zoom, Google Meets, and Microsoft Teams; patient portals; chat boxes; webinars; and wearable devices [12]. Telerehabilitation and telehealth for self-management have been shown to be at least equivalent to in-person care [13-16]. Telehealth services provide several conveniences, including lower costs associated with individual visits. Helplines offer a highly accessible route to determine appropriateness and to promote accessibility of telerehabilitation [17]. Webinars represent a technology that provides the opportunity to engage with a variety of audiences to provide education, information, and support [18].

In order to assess whether the telehealth modalities adopted during the pandemic are efficient and effective at meeting the needs of the populations they serve, we need to employ the most relevant and rigorous evaluation modalities. Many studies have evaluated telehealth and telerehabilitation services [13,18-30]. A 2017 systematic review (n=137 articles) analyzed the successful evaluation methodologies utilized by telehealth projects and indicated that evaluation studies measured the quality of telehealth services through patient and provider satisfaction, economic benefit, and clinical outcomes [23]. Other studies mentioned similar outcome measures of interest for telehealth evaluations including patient and provider satisfaction [24,25]; cost-effectiveness [18-22,24-30]; adherence to, and communication of, care advice [24,25]; and timeliness and responsiveness of the service [25]. An evaluation specifically assessing a nurse-led telehealth line concluded that calling the line resulted in a change in the care management of patients [27]. These changes translated into a reduction in system-level health care costs [27]. A webinar-focused evaluation found that 90.1% of participants indicated that the webinar content was relevant to their interests and needs [18]. Knowledge, awareness of available resources, and confidence to discuss webinar topics increased after attending the webinar [18]. These studies can inform the evaluation approaches utilized to assess the novel telehealth modalities adopted during the pandemic.

### Organizational Context

Rehabilitation aims to “enhance function for meaningful living” by focusing on the impacts that disease and disability have on function [13]. Rehabilitation is described as “…integral to all aspects of health and well-being, from health promotion and prevention to end-of-life care and across the lifespan” [13]. Rehabilitation is often an interprofessional collaboration including patients, families, and diverse disciplines [13].

Community rehabilitation and psychosocial care capacity has been severely challenged by the pandemic. In addition, the spinal cord injury community in Alberta, Canada expected that the disruption of services due to the pandemic would cause more hospitalizations and emergency room visits of persons with lived experience. In response to inconsistencies in health service delivery and variable social distancing mandates, community rehabilitation stakeholders and members of the spinal cord injury community came together in the spring of 2020 to create novel solutions as follows: Rehabilitation Advice Line (RAL) and Alberta Spinal Cord Injury Community Interactive Learning Seminars (AB-SCILS). Multimedia Appendix 1 provides further details on both initiatives including the services provided, operating logistics, and target audience. In general, the RAL is a telephone advice line the helps to eliminate geographical issues with access and provide much needed advice to address the rehabilitation concerns of those living in the community during the COVID-19 pandemic. The AB-SCILS is a monthly webinar series aimed at improving the audience’s knowledge about spinal cord injury, as well as empowerment and management strategies of persons with lived experience and their families.

### Research Aim and Objectives

The aim of this protocol is to assess the short- and long-term impacts and sustainability of the RAL and AB-SCILS. We are interested in understanding the impact of the RAL on individuals with neurological conditions, particularly spinal cord injury, to correspond with the outcomes of the AB-SCILS evaluation.

The RAL program evaluation has the following three specific objectives to clarify impact and feasibility: (1) To clarify the population that uses the RAL in the first 6 months of operation (including personal and geographical demographics, and clinical conditions); (2) To understand the association between RAL use and patient-reported outcomes; and (3) To understand the impact, if any, on health service utilization after RAL use (including emergency room visits, hospitalizations, and primary care visits).

The AB-SCILS evaluation has the following three main objectives to clarify impact and sustainability: (1) To clarify the population attending AB-SCILS during a 6-month period; (2) To evaluate and understand the impact of the initiative on social connectedness, perceptions of disability, and overall quality of life (ie, for people with spinal cord injury) and interactions (for families, service providers, and others); and (3) To explore the long-term sustainability of the initiative.

## Methods

### Overview

For both the RAL evaluation and the AB-SCILS evaluation, we will use an explanatory mixed-methods design [31]. These methods will include secondary data analyses, surveys, and interviews. For the RAL evaluation, we will also conduct narrative analyses of calls using artificial intelligence (AI) and machine learning (ML). AI/ML analysis will be used to provide deeper insights into data recorded during calls to the RAL to provide a more comprehensive analysis of the call data. For the AB-SCILS evaluation, we will also use a collaborative community engagement strategy to ensure that we are held accountable to the spinal cord injury community and to get real-time feedback on AB-SCILS. We have been in consultation with four persons with lived experience with spinal cord injury, and we want to ensure that this process is sustained. We also want to ensure that we are held accountable for the suggestions that these community members provide. As a result, we will be hosting bimonthly focus group discussions with these four members of the spinal cord injury community. The intentions of these focus groups will be to report back to the spinal cord injury community on how their input has been incorporated into the AB-SCILS, therefore working within the “involvement” stage from the community-engagement framework [32]. We will also be continually looking for new input and suggestions on how we can improve the AB-SCILS in the future.

**Figure 1:**
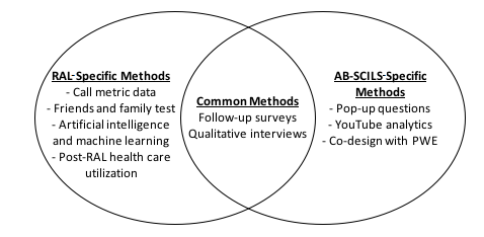
Common and unique methods utilized by each evaluation. AB-SCILS: Alberta Spinal Cord Injury Community Interactive Learning Seminar; PWE: persons with lived experience; RAL: Rehabilitation Advice Line.

The RAL and AB-SCILS evaluation methodologies are each separated into three sections based on specific evaluation aims. Figure 1 outlines the common and unique methods utilized by each evaluation to ensure readability and clarity.

### RAL Evaluation Method

#### Study Population

The study population includes callers to the RAL since its launch on May 12, 2020.

To be included as a caller in the study, participants must be 18 years of age or older and must receive advice through the RAL for neurological, musculoskeletal, and post-COVID-19 with rehabilitation concerns. They must also be able to read and understand English on their own or have support from their own family or friends. There are no exclusion criteria for this study.

#### Evaluation Aim 1: To Understand the Population Using the RAL

##### Recruitment

Our provincial health care system (Alberta Health Services) provides capabilities to obtain real-time data from its electronic medical records (ie, ECHO and the Tableau Dashboard), as well as call metric data through a cloud-based software (ie, Genesys Purcloud). This way, the RAL leadership is able to monitor productivity, clinical content discussed on the call, and caller demographics, allowing them to troubleshoot with clinicians to ensure the safety and quality of the service.

##### Data Collection

RAL data will be retrieved from the Tableau dashboard and Genesys by one of the study team members. Data will be downloaded into a password-protected Excel spreadsheet and shared between team members by SharePoint.

Table 1 describes the data we will retrieve for all of the calls made during the 6-month post-launch period. Call metrics (ie, offered calls, abandoned calls, and call hold duration) will also be retrieved for data analysis.

**Table 1:** Variables of interest for secondary data analysis.

Category	Variable for secondary data analysis
Caller/patient variables	1. Caller age 2. Caller gender
Call metrics	1. Number of distinct callers 2. Number of calls in total and per week 3. Number of call backs in total and per week 4. Average call length in total and per week 5. Number of abandoned calls in total and per week 6. Average call hold time in total and per week

##### Sampling

All data points will be included in the calculations. This will allow us to get a clear picture of the population utilizing the RAL service in the first 6 months after launch. Extrapolating from the data collected in the first month following the RAL launch, it is likely that we will have data on approximately 1200 RAL calls (approximately 200 callers per month for 6 months).

##### Data Analysis

Data will be analyzed using SPSS statistical software (IBM Corp). Data will be used to calculate descriptive statistics on the individuals who call the RAL in the 6 months after launch. We will calculate normality as well as the mean and standard deviation of any interval data analyzed such as caller age. We will also calculate the frequency of any categorical data analyzed such as caller gender and disposition of calls. We will look for associations between variables using the chi-square test and regression analysis. We may perform a time trend analysis on the descriptive data.

#### Evaluation Aim 2: To Understand the RAL Call Experience

To understand the RAL call experience, we will conduct interviews and surveys, as well as use ML. The surveys include patient-reported experience and outcome measures, particularly the friends and family test at the end of each RAL call and study-specific 3-month follow-up surveys with RAL callers. The interviews will be with RAL callers. We will analyze the RAL clinician clinical notes for each call using ML.

##### Friends and Family Test

###### Recruitment

The friends and family test will be administered to every RAL caller at the end of the call. We will conduct secondary data analyses of the results when we perform the data analysis described above. As part of standard practice, participants can choose to answer the question or not answer the question.

The friends and family test has been validated in the United Kingdom National Health Care System and is a direct test, which, when combined with follow-up questions, provides a tool to identify good and bad performance and encourage staff to make improvements when necessary [33]. The specific question is as follows: “Would you recommend this service to a friend or family member?” The response is yes or no.

###### Data Collection

Data are captured in our provincial medical records (ie, ECHO and the Tableau Dashboard) during every RAL call interaction as described above.

###### Sampling

All data collected through the administration of the friends and family test will be included in the calculations. This will allow us to get a clear picture of the population utilizing the RAL service in the first 6 months after launch. We expect approximately 1200 responses to the friends and family test at 6 months after launch. This number is an extrapolation based on data collected in the first month following the RAL launch (approximately 200 callers per month for 6 months).

###### Data Analysis

Data analysis will be completed using SPSS statistical software at 6 months after launch (see above). We will calculate the frequency of “yes” and “no” responses collected during the friends and family test. Results of the friends and family test will be compared and analyzed with patient demographics calculated earlier to see if the results are associated.

##### Follow-Up Surveys With RAL Callers

###### Recruitment

All RAL callers who consent to future contact will be offered the opportunity to complete the follow-up surveys at the 3-month time point following their call. A research assistant will go through the call memos for each month to identify which callers consented for future contact. This list will be cross-referenced with the email list from the RAL. Callers’ emails are added to the list if they are sent resources following the call. For the callers whose email addresses are available, we will send the survey link by email. For the callers whose email addresses are not available, we will call them to ask them if they would be interested in participating in the survey. If they say yes, we will ask for their email addresses and send the survey link. Callers will be sent the survey 1 to 3 months following their call to the RAL. We aim to recruit approximately 30% of all RAL callers for each month. Extrapolating from data collected during the first month after launch, 30% of callers would be approximately 60 callers a month or 180 callers over 3 months.

As part of standard practice, RAL clinicians ask callers if they consent to a follow-up call in a week. It is also standard practice for Alberta Health Services to send out follow-up satisfaction surveys. RAL callers will be free from coercion to participate since surveys will be administered by email and they can choose whether to complete the surveys at their discretion. We will consider the completion of surveys as implied consent.

###### Data Collection

A research assistant will be responsible for sending out the emailed follow-up surveys. Survey responses will be captured by the Alberta Health Services instance of REDCap. Each participant will receive automatic 1-week and 2-week reminder emails if the survey has not been completed through REDCap. The surveys of interest will take approximately 5 to 10 minutes for a caller to complete. The suite of surveys aims to capture how callers are doing 1 to 3 months following their call to the RAL, their perception of the RAL, and general demographic information. All developed surveys are considered valid and reliable [34-42].

- *Patient experience with telephone health line service*: The RAL Patient Experience Survey has been adapted with permission from the Health Link Patient Satisfaction Survey. The Health Link survey was a province-wide initiative conducted by Health Link staff for callers who contacted Health Link between April 1, 2013, and March 31, 2014 [41]. Survey objectives include measuring consumer satisfaction with Health Link and assessing Health Link’s impact on users’ health knowledge and whether they followed through with nurses’ advice [41]. Adapting this existing survey will allow for comparisons with the Health Link service.
- *Patient quality of life*: The EuroQol 5 Dimension 5 Level (EQ-5D-5L) is a standardized generic measure of health status that consists of two pages as follows: the EQ-5D-5L descriptive system and the EuroQol Visual Analog Scale (EQ-VAS) [40]. The EQ-5D-5L descriptive system contains five levels of severity for the following five dimensions: mobility, self-care, usual activities, pain/discomfort, and anxiety/depression [40]. A resultant five-digit number, with each number expressing a dimension, describes the respondent’s health state [40]. The EQ-VAS records the respondent’s self-rated health on a 20-cm vertical visual analog scale, with endpoints of “the best health you can imagine” (score 100) and “the worst health you can imagine” (score 0) [40]. Convergent validity of the 5L version of the EQ-5D appeared with the WHO-5 Well-Being questionnaire as all Spearman rank order coefficients were significant (*P*<.001) and ranged in value across the various dimensions of the 5L versus WHO-5 from 0.33 to 0.61 [40].
- *Self-efficacy to manage chronic health conditions*: The Self-Efficacy for Managing Chronic Disease-6 (SEMCD-6) scale is a six‐item scale measuring respondents’ confidence in their ability to manage fatigue, pain, emotional distress, and other symptoms; to do things other than take medication to reduce illness impact; and to carry out tasks and activities that may reduce the need to see a doctor [37]. Respondents are asked to rate their confidence in performing certain tasks regularly at the present time [37]. Items are rated on a numerical scale ranging from 1 (not confident at all) to 10 (totally confident) [37]. The score for the scale is the mean of all items, with higher scores reflecting greater self‐efficacy [37]. A study reviewing eight independent studies investigated the psychometric properties of the scale. It was found that Cronbach alpha was a minimum of .88 across studies, the floor and ceiling effects were minimal, the measure was sensitive to change, and moderate and significant correlations provided convergent validity evidence when measured against selected health indicators [36].
- *Social support*: The Interpersonal Support Evaluation List-12 (ISEL-12) is a short-form version (12 questions) of the traditional ISEL and measures perceived social support [39]. Each question is measured from one to four, with one indicating “definitely false” and four indicating “definitely true” [39]. The ISEL-12 is scored by summing the items to create an overall social support score with high scores indicating high levels of social support [39]. The psychometric properties of the ISEL-12 have been previously determined [39]. The scale has high internal consistency (Cronbach alpha >.7) [39].
- *Telehealth usability questionnaire*: The Telehealth Usability Questionnaire-10 (TUQ-10) was shortened by Alberta Health Service’s Virtual Health team in collaboration with the primary author, Dr B Parmanto [42]. The TUQ-10 measures the following domains: usefulness, ease of use/learnability, interface quality, reliability, and satisfaction/future use [42]. The TUQ-10 has a Cronbach alpha of ≥.8, which suggests it has good internal consistency and reliability [42].
- *Patient demographics*: We will collect participant demographic data to describe the follow-up survey sample. Self-reported demographic data will include age, sex, marital status, education level, current employment status, and ethnicity. All of these data are not collected in the original RAL call, so we will collect them separately. We will also check for associations between these patient demographic factors and other survey responses.

At the end of the follow-up surveys, participants will be asked if they consent to be contacted for an interview at a later date. We aim to recruit eight to 10 participants for follow-up interviews. They will also have the opportunity to enter their name into a random draw for one tablet computer or equivalent.

###### Data Analysis

Descriptive and inferential data analysis will be completed using SPSS statistical software. We will determine frequencies of responses on each of the surveys, patient gender, reason for call, and disposition of call. We will also calculate the mean where it is relevant to do so, for example, patient age. We will conduct analyses to test for associations between survey responses and patient demographics calculated earlier to see if outcomes are associated with particular types of calls, for example.

##### Interviews With Patients

###### Methodological Framework

We will use Sandelowski’s framework for qualitative description. In qualitative description, a researcher does not deliberately choose to describe an event in terms of a specific framework or system but rather presents the facts of the study in layman’s terms [43]. Qualitative description studies often draw their results from naturalistic inquiry or the studying of something in its natural state [43]. This form of study results in “thick” description, which is preferable to “thin” description obtained from solely quantitative research [43]. Data collection in qualitative descriptive studies often focuses on the who, what, and where or events and experiences, and includes moderately structured open-ended interviews [43]. Qualitative content analysis is the analysis method of choice as the goal is to summarize the information within the verbal data [43].

###### Recruitment

We plan to recruit eight to 10 patients to participate in interviews. Patients who complete follow-up surveys will be asked if they consent to be contacted for a follow-up interview, which would be organized by phone in the 4 weeks after their follow-up call. If patients consent to be contacted, their contact information will be shared with the study team. The research team will then follow-up with the participants to arrange a date and time for the phone interview. Patients will be emailed or mailed the study information sheet and consent form prior to their interview.

###### Data Collection

Participants who agree to an interview will be called at their agreed upon interview date and time. Prior to conducting the interview, the interviewer will go over the consent form with the participants to ensure they understand that the interview will be audio-recorded, that they can choose not to answer any questions that they do not want to, and that they can stop the interview at any time without reason, fear, or repercussions.

The interviews will be semistructured and will last approximately 30 to 60 minutes. The interviews will ask about call experiences, perceived successes and challenges of the call itself, and perceived values or suggestion for improvement of the RAL. The interviewer will probe for further details as necessary. Interviews will be audio recorded on a secure device devoted to the study with the participants consent to capture what they say and exactly how they say it. Interview participants will receive a Can $20 gift card for their participation.

###### Data Analysis

Following interviews, audio recordings will be transcribed verbatim and subsequently deleted. Thematic analysis will be completed on the interview transcripts after every three interviews to inform any modifications or different questions to ask in subsequent interviews if deemed necessary during meetings with the research team.

Thematic analysis will be completed on all of the transcripts. Thematic analysis involves reading through the transcripts and grouping similar ideas together as codes. Codes are then later grouped together into overarching themes. One member of the research team will code all of the transcripts using NVivo software (QSR International). To ensure accuracy of coding, another member of the research team will code a portion of the transcripts using NVivo software. The two research team members will meet to discuss codes and develop a common analytical coding framework. The transcripts will then be reread to ensure that they have been coded appropriately based on the agreed upon analytical coding framework.

###### Rigor

Throughout the study, steps will be taken to ensure transferability, credibility, and dependability of the data, therefore enhancing rigor. Transferability refers to how well a study’s findings can be applied in broader contexts [44]. By using a semistructured interview guide as well as probing questions, participants are prompted to provide in-depth answers. The high level of detail in participant answers allows potential knowledge users to gain better insights into how the conclusions of the study were reached and how these findings can be applied to broader situations. Dependability refers to the ability of findings to be replicated in the future [44]. Complete records will be kept throughout the study. This would allow anyone to track study progression in the future if they wish to do so. The credibility of the study will be enhanced by having multiple research team members involved in the coding process and the development of the coding framework. Moreover, audio recordings will be transcribed verbatim so that the researchers can capture what the participants say and exactly how they say it.

##### Analysis of RAL Call Documentation Through ML

###### Data Collection

Data are captured in our provincial medical records (ie, ECHO and the Tableau Dashboard) during every RAL call interaction as described above. Following input into the ECHO platform, data then flows to the Tableau dashboard. Key demographic information about the callers, such as their age and health care zone, call length, and free text clinical notes detailing the patient’s reason for phoning the RAL; rehabilitation assessments; patient concerns; and the information/services provided by the RAL staff to the patient will be analyzed using a novel AI/ML system. The AI/ML system is used to provide caller experience and engagement information through processing the user note data after anonymization.

###### Sampling

Analysis of demographic and free-text note data will be completed on all calls received by the RAL from May 12, 2020, to October 31, 2020. Including all calls in the sample will allow us to get a clear picture of the RAL caller experience during the first 5 months after launch.

###### Data Analysis

**Figure 2:**
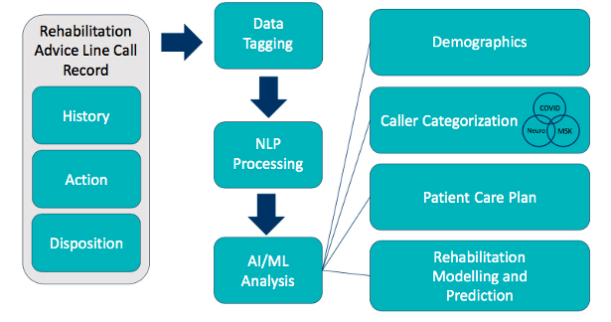
Outline of the analysis to be performed on Rehabilitation Advice Line (RAL) caller data by the combined artificial intelligence (AI) and machine learning (ML) system. MSK: musculoskeletal.

The AI/ML natural language processing (NLP) system is designed as a case study to analyze the effectiveness of the RAL. The AI/ML system will combine and interpret data taken directly from the Tableau dashboard and the information from the free-form clinical notes captured during a call to the RAL. As shown in Figure 2, the processing pipeline for the RAL data system will consist of the following two main components: NLP-based preprocessing of clinical notes and an AI/ML-based system for modeling and analyzing the collected data.

NLP preprocessing will assist with analyzing the information contained in the free-text clinical notes entered into the ECHO platform. The call notes consist of unstructured data that can be classified into the following three broad categories: (1) *History* including previous patient diagnoses, medications, and existing symptoms; (2) *Action* taken by the RAL advisor during the call including discussion of current symptoms (including pain, weakness, difficulty performing activities of daily living, etc), subjective over-the-phone assessment, and cause of the condition (eg, if it was caused through injury); and (3) *Disposition* detailing the advice provided or service referrals given to the patient. By capturing this information, the RAL provides a means of monitoring and providing assistance to individual patients.

As an example of the analysis to be performed, the AI/ML system allows for a more meaningful interpretation of the RAL phone conversation by considering factors such as the frequency certain keywords are mentioned (such as patient-reported conditions or symptoms, comparison of keywords between different callers and geographic areas, and finding correlations between topics discussed in each call). The AI/ML system distills this analysis down into a list of common conditions, symptoms, and reasons for calling the RAL to get a metric of the underlying reasons persons are phoning into the RAL, what assessment was undertaken during the call, and what disposition was offered by the RAL. Along with traditional evaluation metrics that will be collected during patient surveys and interviews, the AI/ML NLP system allows for more in-depth understanding of the needs and services required and provided for persons phoning into the RAL. The system allows for automatically capturing demographic data by categorizing the reason for the call as resulting from musculoskeletal, neurological, COVID-19, or other conditions and analysis of the disposition to better understand the patient care plan.

Caller demographic information, including caller age, call length, and caller zone, retrieved from the ECHO platform will be used as part of the AI/ML analysis (see Multimedia Appendix 2 for further details).

An NLP tool will be used to analyze the clinical notes taken during the call and this information, in addition to the demographic information, will be used within the AI/ML system. The NLP tool works on the free-form text recorded by the RAL clinician, and will be used to identify medically relevant keywords and phrases within the call notes. The NLP analysis will be used to determine and categorize the reason for the call, caller’s medical history, assessment, and disposition (see Multimedia Appendix 3 for further details).

#### Evaluation Aim 3: To Understand Health Service Utilization After the RAL

##### Data Collection

Patient personal health numbers are also captured by the provincial electronic medical record (ie, ECHO) during all RAL call interactions. With support from data analytics in Alberta Health Services, patient personal health numbers will be followed to determine how patients utilized health care services following the RAL encounter. The National Ambulatory Care Reporting System will be used as a source of data by the data analytics team in Alberta Health Services. The data analytics team will retrieve the data and share it in the form of a password-protected Excel spreadsheet with the research team through Alberta Health Services email to ensure we are working behind a firewall.

##### Sampling

All caller data will be included in the calculations to clarify how RAL callers are utilizing health care services following their call within the first 6 months of the RAL being available.

##### Data Analysis

Data will be analyzed using SPSS statistical software. Data will be used to calculate descriptive statistics on the health service utilization after the RAL launch of individuals who called the RAL in the 6 months after the launch. We will calculate the average frequency of emergency room visits before and after the RAL call. We will calculate the number of unique patients who visited the emergency room before and/or after their RAL call. We will also calculate the average number of days before and/or after someone visited the emergency room with respect to when they called the RAL and use that to calculate the mean difference (mean days after minus mean days before).

### AB-SCILS Evaluation Method

#### Study Population

In consultation with persons with lived experience with spinal cord injury, we have determined that the AB-SCILS should not be solely targeted toward persons with lived experience since those who have lived with spinal cord injury for many years know a lot of the information being shared in the webinars. To build a broader spinal cord injury community that is equipped with appropriate knowledge to work with persons with lived experience, the AB-SCILS will target all persons with lived experience, including hospitalized patients and their families, health care professionals who are part of the spinal cord care journey (including general practitioners, nurses, rehabilitation experts, social workers, and physiatrists), and members of the broader community (ie, teachers, students, and city designers).

The study population includes attendees to the AB-SCILS. This population will be quite diverse and variable as AB-SCILS evolves. A detailed demographic analysis will be one of the factors we address in our evaluation. Attendees will likely include individuals living with spinal cord injury and their family members, health care professionals, and members of the broader community like teachers.

Participants must be 18 years of age or older and have attended at least one AB-SCILS session. They must also be able to read and understand English on their own or have support from their own family or friends. There are no exclusion criteria for this study.

#### Evaluation Aim 1: To Understand the Population Attending the AB-SCILS

##### Demographics

###### Data Collection

Patient demographics will be collected via pop-up questions at each webinar during the evaluation period (from November 2020 to April 2021). Demographics will include the following: (1) affiliation or interest in the webinar (person with spinal cord injury, family member of someone with spinal cord injury, health care provider, researcher, program manager, student, or other); (2) general location (urban, rural, or metropolitan center); (3) whether attendees are from Edmonton, Calgary, elsewhere in Alberta, or outside of Alberta; (4) attendance record (is this their first webinar, have they attended one to three webinars, have they attended four to six webinars, or have they attended more than six webinars); (5) whether they have accessed past webinars after the live session; (6) preferred availability for future webinars; and (7) preferred topics for future webinars.

###### Sampling

All demographic information collected during the 6-month evaluation period will be included in the evaluation to help us understand the population attending the AB-SCILS.

###### Data Analysis

We will calculate the frequency of responses for each of the pop-up questions to help describe the population attending the AB-SCILS. We will also perform chi-square tests to test for associations between variables if our data meets all of the assumptions of this test.

##### YouTube Analytics

###### Data Collection

We will clarify the demographics of those accessing the AB-SCILS through YouTube after the live session. We will pull data on where individuals are viewing the videos from (YouTube provides a general location), as well as how many unique views each video gets and when the videos are viewed. Data will be accessed through the AB-SCILS YouTube account. Aggregate data will be pulled from YouTube by a research assistant acting behind the Alberta Health Services firewall.

###### Sampling

We will analyze all YouTube analytics data collected during the 6-month evaluation period. All data contained in YouTube analytics are aggregate data, so we will be able to get a general picture of who is accessing the AB-SCILS after the live session.

###### Data Analysis

The analysis will include frequency calculations of where individuals are viewing the video from, as well as how many unique views each video gets and when. We will also perform chi-square tests to test for associations between variables if our data meets all of the assumptions of this test. All of these data will be analyzed on a video-to-video basis following the completion of the evaluation period.

#### Evaluation Aim 2: To Understand the AB-SCILS Webinar Experience

##### Recruitment

All AB-SCILS attendees will be offered the opportunity to complete follow-up surveys. Our goal is to have at least approximately 30% of all AB-SCILS attendees complete the follow-up surveys. It is possible that more attendees may fill out the follow-up surveys, and this additional data would be welcome as we seek a convenience sample. Attendees are able to complete the surveys more than once during the 6-month evaluation period and are encouraged to do so by having their names entered into a draw for a gift card following each webinar during the evaluation period, as well as a draw for a tablet computer at the end of the project. Having attendees complete the survey every month for 6 months would be ideal, as this would allow us to do some longitudinal analyses of the data. However, we recognize the infeasibility of every attendee completing surveys after each AB-SCILS session. We will also examine the data cross-sectionally to get a snapshot of attendee perspectives with the AB-SCILS.

##### Data Collection

Participants will have the opportunity to click the survey link during and after each webinar. Survey responses will be captured by the Alberta Health Services instance of REDCap.

The survey package has been co-developed with persons with lived experience. This survey package includes questions about the ability of the AB-SCILS to foster social connectedness and change perceptions about disability. The surveys will also include more specific demographic questions such as age, gender, and educational background. The demographic information will be used to complete correlational analyses during data analysis. The surveys of interest will take approximately 10 to 15 minutes for an AB-SCILS attendee to complete, and informed consent will be implied with the return of fully or partially completed surveys.

At the end of the follow-up surveys, participants will be asked if they consent to be contacted for an interview at a later date. We aim to recruit eight to 10 participants for follow-up interviews.

##### Data Analysis

Descriptive and inferential data analyses will be completed using SPSS and Stata software (StataCorp). We will calculate frequencies of responses on each of the surveys where appropriate. We will also calculate the mean where it is relevant to do so. We will conduct chi-square analyses to test for associations between survey responses and patient demographics. We will perform regression analysis to test for statistically significant relationships between survey responses and demographic data.

#### Evaluation Aim 3: To Understand the Long-Term Sustainability of the AB-SCILS

##### Methodological Framework

We will utilize Sandelowski’s framework for qualitative description to ground our qualitative work as discussed above [43].

##### Recruitment

We plan to recruit approximately eight to 10 AB-SCILS attendees, who have attended at least one AB-SCILS, to participate in interviews. Individuals who complete follow-up surveys will be asked if they consent to be contacted for a follow-up interview, which would be organized by phone in the 4 weeks after their follow-up call. If patients consent to be contacted, their contact information will be shared with the study team. The research team will then follow-up with the participants to arrange a date and time for the phone interview. Patients will be emailed or mailed the study information sheet and consent form prior to their interview.

##### Data Collection

Interview data collection for the AB-SCILS portion of the study will follow the same process as outlined above in the RAL evaluation methods. The interviews will ask about webinar experiences, perceived successes and challenges of the webinars themselves, and some takeaways from the webinars (ie, whether their views about spinal cord injury were challenged and how so), as well as the ability of the AB-SCILS to build a broader spinal cord injury community. Questions will be related to understanding the experiences of attendees in respect to the AB-SCILS, for example, “Could you describe your experience with AB-SCILS?” and “Did attending the AB-SCILS challenge your assumptions about what *normal* life with spinal cord injury could look like?”

##### Data Analysis

Following interviews, audio recordings will be transcribed verbatim by a research assistant and subsequently deleted. Thematic analysis will be completed on all of the transcripts as described above in the RAL evaluation methods. However, thematic analysis for the AB-SCILS evaluation will use Dedoose software (SocioCultural Research Consultants) rather than NVivo as described above.

##### Rigor

The same steps outlined above will be taken to ensure transferability, credibility, and dependability of our qualitative work, therefore enhancing rigor.

## Results

At the time of protocol submission, we have completed qualitative interviews with 10 RAL callers and received 68 survey responses to the RAL follow-up surveys. Due to the integrative approach of our methods, we are currently completing all analyses to accumulate our findings to address the research questions.

For the AB-SCILS, we have begun administering surveys and have collected 25 survey responses at the time of protocol submission. We have also completed five qualitative interviews. We will continue to collect survey responses in the upcoming three webinars. Following each of the upcoming three webinars, we hope to interview two attendees to gain a more in depth understanding of their experience. Data analysis will commence shortly after the completion of data collection.

## Discussion

### Preliminary Gap Addressed

The study outlined in this protocol will help us learn how best to advance virtual care in Alberta by clarifying impact and feasibility, and informing sustainability of telehealth modalities. The pandemic has fostered the adoption of telehealth practices to improve the continuity of care for people with disabilities. Several studies have aimed to measure the impact and feasibility of telehealth initiatives adopted during the pandemic [12,45,46]. However, very few evaluation studies about telehealth initiatives adopted during the pandemic have been able to be carried out due to social distancing mandates, global lockdowns, and the reallocation of resources to aid in the pandemic response. The study discussed in this protocol will help to address this gap and will contribute to the knowledge base on the impact, feasibility, and sustainability of telehealth initiatives during and after COVID-19.

### Study Strengths

There are many factors that contribute to the strength of our proposed work. Implementing and evaluating different virtual modalities synchronously allows for the building of knowledge on the complementarity of these methods. This creation of knowledge provides an opportunity to improve each modality in order to synergize the positive effects of each. Both portions of the proposed study have unique qualities that contribute to the novel nature of the evaluation as a whole and will aid in learning how to best advance virtual care in Alberta. Bridging mixed-methods research with AI/ML modalities can assist in understanding the utility of such technologies when health care resources are being strained due to the pandemic [47]. Telehealth initiatives supplemented with AI/ML methods have been shown to be particularly useful for quality improvement for existing clinical practice and service delivery [48]. However, recent literature in telemedicine is still offering new potential applications for these methods, which suggests that their full potential for utility has not yet been attained [49]. Utilizing AI/ML technologies in our current work is justified as it will help contribute to the knowledge base of evaluating telehealth strategies with these technologies.

The co-design of the AB-SCILS webinar series also presents a unique opportunity. Previously published literature on patient engagement and co-design of health care services has demonstrated the need for strategies to build strong relationships between patients and health care professionals to ensure good communication and ultimately achieve positive outcomes [50]. The proposed study outlines one such strategy, and its overall effectiveness will contribute to the potential success of the evaluation. After the initial engagement with community members, they decided to continue working with us in the “involvement” category after discussing the IAP engagement framework [32]. This is an example of the diverse expectations and views from community members and shows that for some groups, being “empowered” or participating as equal partners in decision making is not possible due to their capacity to be engaged in such an intense manner. There is this trend to think that all community-based work has to be at least at the “collaboration” level if not the “empowerment” level [32]. We are demonstrating in this study that this is not the case as the members of the community did not feel they had the capacity or time to be engaged at the higher levels of the spectrum. We want to highlight that being “involved” versus “empowered” does not mean that the engagement is not meaningful. We as researchers must still ensure that we are held accountable for the suggestions that these community members provide and react accordingly.

### Challenges and Limitations

We have faced challenges to date with our research. Conducting the RAL evaluation during the COVID-19 pandemic has not occurred without challenges. We have collected survey data for approximately 4 months and had 68 survey responses, while the RAL had 537 clinical call interactions during the first five and a half months of operation. The low survey responses are due to challenges in survey recruitment. We initially planned on collecting survey data by sending the survey link via email to RAL callers. However, we soon realized that we did not have many RAL caller email addresses since not all callers get resources sent to them after the call interaction. Further, not all RAL callers consented to be contacted about their call experience or did not want to participate in the survey due to their call interaction being months prior. Of the 537 clinical call interactions collected during the study period, only 304 had caller consent for follow-up contact. Through the RAL email list and by calling RAL callers, we were able to send the survey link to 162 individuals. These values represent a 42% response rate, which we consider satisfactory given the challenges associated with recruitment.

There are also potential limitations that go along with our proposed methodology. While the evaluation methods for both modalities are parallel in nature, bringing the results together may be challenging as the methods are not exactly the same. This may limit our ability to apply the learnings from each evaluation separately to improve our understanding of the effect of virtual health modalities in Alberta as a whole. To overcome this challenge, we continue to have monthly meetings with team members from both evaluations to share our learnings and inform our work moving forward, so that all parties understand how both evaluations complement one another. Our survey responses and qualitative interview data collected for the RAL evaluation may also have the issue of recall bias since we did not contact participants until 3 months after their call interaction. Participants may not have remembered important details of their call interaction, which may limit the quality of the data collected. However, the timing of survey administration and interviewing was deliberately chosen as we were interested in understanding how callers were doing 3 months following their interaction. If we would have administered the survey immediately following their call interaction, we would not know how the participants are doing in the longer term. The dual modality of the proposed study may offer a strategy to overcome this limitation since the AB-SCILS surveys and interviews will occur immediately following each webinar. We will be able to understand the short-term effects of an interaction with a virtual modality from the AB-SCILS evaluation and the longer-term effects from the RAL evaluation.

### Conclusion

Understanding the impact and sustainability of the proposed telehealth modalities is important. The results of the evaluation will provide data that can be actioned and serve to improve other telehealth modalities in the future, since health systems need this information to make decisions on resource allocation, especially in an uncertain pandemic climate. Evaluating the RAL and AB-SCILS to ensure their effectiveness demonstrates that Alberta Health Services and the health system care about ensuring the best practice even after a shift to primarily virtual care.

## Appendix

Multimedia Appendix 1 Detailed explanations of the Rehabilitation Advice Line (RAL) and Alberta Spinal Cord Injury Community Interactive Learning Seminars (AB-SCILS).

Multimedia Appendix 2 Demographic information for artificial intelligence and machine learning analyses.

Multimedia Appendix 3 Natural language processing categories.

Multimedia Appendix 4 Peer-review reports.

Multimedia Appendix 5 Authors' responses to peer-review report comments.

## Abbreviations

AB-SCILS: Alberta Spinal Cord Injury Interactive Learning Seminars
AI: artificial intelligence
EQ-5D-5L: EuroQol 5 Dimension 5 Level
EQ-VAS: EuroQol Visual Analog Scale
ISEL-12: Interpersonal Support Evaluation List-12
ML: machine learning
NLP: natural language processing
RAL: Rehabilitation Advice Line
TUQ-10: Telehealth Usability Questionnaire-10
