# Supplementary material for: Evaluating Community-Facing Virtual Modalities to Support Complex Neurological Populations During the COVID-19 Pandemic: Protocol for a Mixed Methods Study
Source: JMIR Res Protoc. 2021 Jul 23;10(7):e28267. doi: 10.2196/28267 (PMC8315160; doi:10.2196/28267)
Supplement: Multimedia Appendix 1 [file resprot_v10i7e28267_app1.doc]

**Multimedia Appendix 1.** Detailed explanations of the Rehabilitation Advice Line (RAL) and Alberta Spinal Cord Injury Community Interactive Learning Seminars (AB-SCILS).

#### *The RAL*

#### The RAL is a telephone advice line the helps to eliminate geographical issues with access and provide much-needed advice to address the rehabilitation concerns of those living in the community during the COVID-19 pandemic. The RAL provides wayfinding and self-management advice to Albertans with physical conditions related to an existing musculoskeletal or neurological condition or post-COVID-19 recovery needs. The RAL also provides access to social support to address rehabilitation and recovery needs.

The RAL is a collaboration between health system leadership from multiple provincial teams related to bone and joint health, neurosciences, vision, and community rehabilitation. The RAL operates five days a week from 9am–5pm and callers looking for assistance outside those hours are directed to available support via Health Link®, which is a nurse-manned telehealth line operating 24/7 in Alberta. Clinicians provide wayfinding and tele-triage. Callers may be sent to a provincial health information website for self-management tips and resources as appropriate. Callers may be sent to appropriate community service organizations. Those requiring follow-up receive a call-back from the appropriate healthcare professional within one business day to provide the necessary intervention or linkage to existing programs or services as appropriate.

#### *AB-SCILS*

#### AB-SCILS is a monthly webinar series aimed at improving the audience’s knowledge about spinal cord injury as well as empowerment and management strategies of PWE and their families. To accomplish this aim, AB-SCILS is focused on creating a of sense of community, changing perceptions on disability, and improving social connectedness in order to ameliorate the effects of social isolation during the COVID-19 pandemic.

AB-SCILS is a collaboration between PRAXIS Spinal Cord Institute, Spinal Cord Injury Alberta, health care providers in Edmonton and Calgary, and PWE. PRAXIS Spinal Cord Institute is a not-for-profit organization leading and supporting global collaboration in spinal cord injury research, innovation, and care [32]. Spinal Cord Injury Alberta aims to empower PWE to achieve independence and full community participation [33].

The webinar is co-designed and co-presented by PWE and clinical experts so that both perspectives are shared during AB-SCILS. Each webinar topic is based on one of four pillars: medical, research, community living, or active living. The medical pillar is focused on the most common impairments faced by people with spinal cord injury as well as on relevant topics to promote health and prevent medical complications. The research pillar is focused on disseminating scientific investigations directed to improve the lives of people with spinal cord injury. The active living pillar is focused on topics that promote healthy behaviours and prevent physical and mental health problems in the community. The community living pillar is focused on topics that support participation in a meaningful life in the community.

Each webinar is scheduled for 1.5 hours. The presentation portion of the webinar should last no longer than 30 minutes. The presentations are followed by a break-out room session and a question and answer period. For the community living and active living pillars, the break-out room session occurs prior to the question and answer period. For the medical and research pillars, the break-out room session occurs after the question and answer period.

All webinars are recorded and posted on YouTube and are open to the public. The targeted audience is composed of PWE, family members, and service providers involved in their lives.
