# Supplementary material for: Evaluating Community-Facing Virtual Modalities to Support Complex Neurological Populations During the COVID-19 Pandemic: Protocol for a Mixed Methods Study
Source: JMIR Res Protoc. 2021 Jul 23;10(7):e28267. doi: 10.2196/28267 (PMC8315160; doi:10.2196/28267)
Supplement: Multimedia Appendix 2 [file resprot_v10i7e28267_app2.doc]

**Multimedia Appendix 2.** Demographic information for artificial intelligence and machine learning analyses.

- **Caller Age** – The actual caller’s age if a person was phoning in for themselves, or the age of the person that the call was about if another person phoned in on their behalf.
- **Call Length** – Total amount of time the caller spent during the phone conversation with the RAL clinician.
- **Caller Zone** – The actual zone in which the caller lives. This is to break down the analysis into the three healthcare zones (Calgary zone, Edmonton zone, and the combined Rural zones)
