# Supplementary material for: Evaluating Community-Facing Virtual Modalities to Support Complex Neurological Populations During the COVID-19 Pandemic: Protocol for a Mixed Methods Study
Source: JMIR Res Protoc. 2021 Jul 23;10(7):e28267. doi: 10.2196/28267 (PMC8315160; doi:10.2196/28267)
Supplement: Multimedia Appendix 3 [file resprot_v10i7e28267_app3.doc]

**Multimedia Appendix 3.** Natural language processing categories.

- **Reason for Call** – The reason for the call to the RAL, including categories such as wayfinding for services cancelled due to COVID-19, acute injury rehabilitation, and post-COVID-19 rehabilitation needs.
- **Medical History** – The caller’s relevant medical history, including existing chronic or acute musculoskeletal, neurological, or other conditions.
- **Assessment** – The formal and informal assessment provided by the RAL clinician, including activities of daily living, standard rehabilitation assessment metrics, and social conditions, and mental health concerns.
- **Disposition** – The care plan and services provided by the RAL clinician, including service referrals, scheduling follow-up phone calls/emails, referral to online information (e.g. AHS website).
