# Supplementary material for: Evaluating Community-Facing Virtual Modalities to Support Complex Neurological Populations During the COVID-19 Pandemic: Protocol for a Mixed Methods Study
Source: JMIR Res Protoc. 2021 Jul 23;10(7):e28267. doi: 10.2196/28267 (PMC8315160; doi:10.2196/28267)
Supplement: Multimedia Appendix 4 [file resprot_v10i7e28267_app4.pdf]

Dear Dr. Loyola-Sanchez,

Thank you for responding to our request for applications for the Praxis Spinal Cord Injury (SCI) COVID-19 Award. We received eleven applications which demonstrated the commitment of our SCI community to improving the care, health, and wellness for those living with SCI during this pandemic.

We were fortunate to work with a cadre of exceptional reviewers who took the time to provide insightful feedback. We are delighted to inform your application entitled "**Evaluation of a Provincial Telehealth Strategy to Promote Community Connection and Improve Access to Rehabilitation Services for Persons with SCI Amidst the COVID-19 Pandemic: An Albertan Collaborative Webinar & Rehabilitation Advice Line Initiative**" was ***provisionally accepted*** for a maximum of $15,000 CAD in funding, subject to review and approval of your written response to the reviewers' comments below, due to funding@praxisinstitute.org no later than eight business days from the date of this letter.
The reviewers had the following comments:

1. Given the focus of the Praxis SCI COVID-19 Award is to create and share COVID-19 related resources with the SCI community, please elaborate on:
   a. What resources from your project will be made available (e.g. from the RAL, Alberta-SCILS) for sharing in the short-term, and
   b. Your dissemination plan to share learnings from your project with the SCI community nationally in addition to the conference presentation and publication.

2. Given one of the criteria of the Award is the ability for the resources to be scaled up easily, please elaborate on:
   a. Any quality improvement/learning questions in your evaluation plan, and
   b. Details on capturing and differentiating the impact from the initial urgent need during the peak of the COVID-19 pandemic and from after the initial need has dissipated on the uptake of your RAL and Alberta-SCILS (assessing attribution/incrementality).

Please submit revised sections of the application (Initiative Plan, Anticipate Impact and Evaluation Metrics, Timeline, and Budget, if applicable) along with your response to the reviewers' comments. Please do not hesitate to contact us at funding@praxisinstitute.org if you have any questions. We also ask that you please inform your co-applicants with the outcome of this application.

We look forward to your response.

Sincerely,

Bill Barrable
Chief Executive Officer

Praxis Spinal Cord Institute
6400-818 West 10th Avenue, Vancouver BC V5Z 1M9
t 604 827 1652
praxisinstitute.org

PRAXIS
Spinal Cord Institute
