# Supplementary material for: Evaluating Community-Facing Virtual Modalities to Support Complex Neurological Populations During the COVID-19 Pandemic: Protocol for a Mixed Methods Study
Source: JMIR Res Protoc. 2021 Jul 23;10(7):e28267. doi: 10.2196/28267 (PMC8315160; doi:10.2196/28267)
Supplement: Multimedia Appendix 5 [file resprot_v10i7e28267_app5.pdf]

Evaluation of a Provincial Telehealth Strategy to Promote Community Connection and Improve Access to Rehabilitation Services for Persons with SCI Amidst the COVID-19 Pandemic: An Albertan Collaborative Webinar & Rehabilitation Advice Line Initiative

Praxis SCI COVID-19 Award

Response to reviewers

1. Given the focus of the Praxis SCI COVID-19 Award is to create and share COVID-19 related resources with the SCI community, please elaborate on:

   a. What resources from your project will be made available (e.g. from the RAL, Alberta-SCILS) for sharing in the short-term?

   Response- currently, we are posting all the webinars and the resources created in the Alberta-SCILS in social media platforms controlled and promoted by SCI-AB. For example, our last webinar was on "exercise at home during social isolation" and we created a series of videos showing how to do a basic strengthening, aerobic, and stretching routine at home, which are now available in the "SCI-AB you tube channel". Further clarification has been added to the "proposed initiative" section of the application.

   The RAL was just launched two weeks ago in Calgary, and we will be collecting information related to the most frequent issues and complaints expressed by persons with SCI. Our intention is to understand the most common problems encountered by the SCI community in order to immediately generate virtual support material that can promote their resolution. Further clarification has been added to the "proposed initiative" section of the application.

   In addition, as we briefly mentioned in the "proposed initiative" section of the application, we are implementing a strategy to cross-share the resources created in the AB- SCILS and RAL. The RAL will be promoted during the upcoming webinars in the AB-SCILS, aiming to increase its utilization by the SCI community. Similarly, we will disseminate the existence of the AB-SCILS with the staff currently attending the phone lines of the RAL as a way to promote this resource among SCI callers. This cross-sharing strategy will also include a constant communication between our provincial telehealth interventions in order to help define relevant topics to include in the AB-SCILS on one hand, and on the other hand suggest the inclusion of relevant support services to the RAL. Further clarification has been added to the "proposed initiative" section of the application.

b. Your dissemination plan to share learnings from your project with the SCI community nationally in addition to the conference presentation and publication.

Response- we plan to communicate and discuss our results through diverse national (e.g. Spinal Cord Injury-Canada, Praxis and the Canadian Spinal Cord Injury Rehabilitation association) and international SCI (e.g. ISCoS) SCI-related organizations' platforms. In addition, we plan to develop a toolkit describing the core processes and evaluation structure for both interventions, which will help replicate the design and implementation of SCI-specific webinars and rehabilitation specific phone services in collaboration with community organizations and peers at national and international levels. We also expect to have the support of Praxis to disseminate our results in diverse peer-led SCI communication platforms throughout Canada. Further clarification has been added to the "dissemination plan" section of the application.

2. Given one of the criteria of the Award is the ability for the resources to be scaled up easily, please elaborate on:

a. Any quality improvement/learning questions in your evaluation plan,

Response- our questionnaires and interview guides will include sections to explore users' satisfaction with the Alberta SCILS and the RAL. In addition, the RAL implementation currently includes an "after action review" that has the purpose of allowing health professionals to reflect and identify technological, clinical and resources challenges immediately after the completion of each phone call. All these strategies will be used to continuously improve the quality of both interventions. Further clarification has been added to the "execution plan" section of the application.

b. Details on capturing and differentiating the impact from the initial urgent need during the peak of the COVID-19 pandemic and from after the initial need has dissipated on the uptake of your RAL and Alberta-SCILS (assessing attribution/incrementality).

Response- our questionnaires and the interview guides will include a specific section to allow users to reflect on the value of the webinars and the phone line beyond the COVID-19 pandemic. Our collaborators with SCI lived experience have pointed out the expectation that both interventions will remain relevant after the pandemic, as they address several service gaps that were present before the COVID 19 pandemic. The conglomerate data analysis on health service utilization related with

the RAL will also include a comparative temporal analysis to attribute the intervention's impact beyond social isolation times. Further clarification has been added to the "analysis" section of the application.

Please submit revised sections of the application (Initiative Plan, Anticipate Impact and Evaluation Metrics, Timeline, and Budget, if applicable) along with your response to the reviewers' comments. Please do not hesitate to contact us at funding@praxisinstitute.org if you have any questions. We also ask that you please inform your co-applicants with the outcome of this application.
